# Describing the initial results of a pragmatic, cluster randomized clinical trial to examine the impact of a multifaceted digital intervention for the prevention of type 2 diabetes mellitus in the primary care setting: intervention design, recruitment strategy and participants’ baseline characteristics of the PREDIABETEXT trial

**DOI:** 10.3389/fendo.2025.1524336

**Published:** 2025-03-31

**Authors:** Sofía Mira-Martínez, Rocío Zamanillo-Campos, Narges Malih, Maria Antonia Fiol-deRoque, Escarlata Angullo-Martínez, Rafael Jimenez, Maria Jesús Serrano-Ripoll, José Iván Oña, Aina M Galmes-Panades, Rocío Gómez-Juanes, Jadwiga Konieczna, Jerónima Miralles, Dora Romaguera, María Clara Vidal-Thomasa, Joan Llobera-Canaves, Mauro García-Toro, Elena Gervilla-García, Catalina Vicens, Oana Bulilete, Juan José Montaño, Margalida Gili, Miquel Roca, Antoni Colom, Ignacio Ricci-Cabello

**Affiliations:** ^1^ Health Research Institute of the Balearic Islands (IdISBa), Palma de Mallorca, Spain; ^2^ Research Group in Primary Care and Promotion-Balearic Islands Community (GRAPP-caIB), Palma de Mallorca, Spain; ^3^ Primary Care Research Unit of Mallorca, Balearic Islands Health Service, Palma, Spain; ^4^ Research Network on Chronicity, Primary Care, and Health Promotion (RICAPPS), Instituto de Salud Carlos III (ISCIII), Madrid, Spain; ^5^ Statistical and Psychometric Procedures Applied in Health Sciences (PSICOMEST), Health Research Institute of the Balearic Islands (IdISBa), Palma de Mallorca, Spain; ^6^ Department of Psychology, University of the Balearic Islands (UIB), Palma, Spain; ^7^ Dra. Teresa Piqué Primary Health Care Center, Balearic Health Service, Palma, Spain; ^8^ Research Group on Nursing, Community & Global Health, Health Research Institute of the Balearic Islands (IdISBa), University Hospital Son Espases (HUSE), Palma, Spain; ^9^ Physical Activity and Sport Sciences Research Group (GICAFE), Institute for Educational Research and Innovation (IRIE), University of the Balearic Islands, Palma, Spain; ^10^ CIBER of Physiopathology of Obesity and Nutrition (CIBEROBN), Instituto de Salud Carlos III, Madrid, Spain; ^11^ University Institute of Health Science Research (IUNICS), University of the Balearic Islands, Palma, Spain; ^12^ Department of Medicine, University of the Balearic Islands, Palma, Spain; ^13^ Research Group on Nutritional Epidemiology & Cardiovascular Physiopathology (NUTRECOR), Health Research Institute of the Balearic Islands (IdISBa), University Hospital Son Espases (HUSE), Palma de Mallorca, Spain; ^14^ Son Serra-La Vileta Primary Health Care Center, Balearic Health Service, Palma, Spain; ^15^ Department of Geography, University of the Balearic Islands (UIB), Palma de Mallorca, Spain

**Keywords:** prediabetic state, preventive health services, clinical trial, primary health care, patient recruitment

## Abstract

**Introduction:**

i) to describe PREDIABETEXT, a novel digital intervention for the prevention of type 2 diabetes; ii) to examine the performance of a strategy for virtual recruitment of participants in a trial to assess its impact, and; iii) to determine the baseline characteristics of the enrolled participants.

**Methods:**

We developed PREDIABETEXT in a multistage process involving systematic literature reviews and qualitative research with end users (primary care patients and professionals). We combined multiple virtual strategies (SMS, phone calls, promotional videos) to recruit healthcare professionals and their patients. We collected baseline data from patients (sociodemographic, behavioral and clinical) and healthcare professionals (sociodemographic and professional experience).

**Results:**

The intervention consisted in delivering personalized short text messages supporting lifestyle behavior changes to people at risk of type 2 diabetes; and online training to their primary healthcare professionals. We recruited 58/133 (43.6%) professionals (30 doctors; 28 nurses) from 16 centers. Most professionals (83%) were women [mean (SD) age 49.69 (10.15)]. We recruited 365/976 (37.4%) patients (54.5% women, 59.82 (9.77) years old. Around half (55.3%) presented obesity (BMI ≥25), 65% hypertension, 43.3% hypercholesterolemia, and 14.8% hypertriglyceridemia.

**Conclusions:**

The PREDIABETEX trial successfully recruited a representative sample of patients at risk of type 2 diabetes and their healthcare providers.

## Introduction

Prediabetes is an intermediate state of hyperglycemia with glycemic parameters above normal but below the diabetes threshold. The World Health Organization (WHO) defines prediabetes based on impaired fasting glucose (IFG - fasting plasma glucose 6.1-6.9 mmol/L), impaired glucose tolerance (IGT - 2 h plasma glucose of 7.8-11.0 mmol/L after ingestion of 75 g of oral glucose load), or a combination of the two based on a 2 h oral glucose tolerance test (1).

The global prevalence of IGT in 2021 was 9.1% (464 million) and is projected to increase to 10.0% (638 million) in 2045. The global prevalence of IFG in 2021 was 5.8% (298 million) and is projected to increase to 6.5% (414 million) in 2045 (2). About 25% of adults with prediabetes will progress into T2DM within 3-5 years (3) and on a lifetime scale, 70% of individuals with prediabetes will develop overt diabetes (4). In addition to being more likely to develop diabetes, individuals with prediabetes are generally more prone to develop pathologies such as diabetic retinopathy, neuropathy, nephropathy, and macrovascular complications (5). Further, long-term consequences of diabetes lead to a lower quality of life, a significant increase in healthcare costs, and death (6).

Lifestyle interventions can effectively prevent progression to diabetes (7–9). Effective treatments include the yearlong behavioral lifestyle change program based on the Diabetes Prevention Program (DPP), which decreased type 2 diabetes incidence by 58% over 3 years in the landmark DPP RCT (10). Unfortunately, few people participate in high intensity diabetes prevention programs. Taking the DPP as an example, only a third patients referred to a DPP initiate the program, and among those who initiate, only 32% remain in the program until completion (11) Barriers to participation and retention in this high intensity, face-to-face program include lack of time and inability or unwillingness of participants to commit to a long-term program (12).

Digitally delivered, lower-intensity interventions may contribute to address these barriers. They have the potential to be a low-cost, massively available, and sustainable strategy for health systems to improve population health. Recent systematic reviews examining the use of mobile health interventions among people with prediabetes observed a lack of significant effect on the incidence of T2DM, and inconsistent results in weight loss, body mass index, and waist circumference changes (13, 14). However, this area of research is still in its infancy, and the available evidence of this type of intervention is limited by the small number and methodologic weaknesses of previous trials (15).

To contribute to address this gap, we developed PREDIABETEXT, a new theory-based, multifaceted, digital intervention; and set up a cluster randomized clinical trial to evaluate its impact on diabetes prevention and cardiovascular risk factors.

The aims of this paper are to: i) describe the design, components and structure of the PREDIABETEXT intervention; ii) describe de design and performance a novel virtual participant recruitment strategy, and; iii) report the baseline sociodemographic and clinical characteristics of the recruited participants.

## Material and methods

### Intervention design and development

The process for the development of the PREDIABETEXT intervention was based on the MRC Guidance for the Development of Complex Interventions (16). This study has five phases. Each phase had a variety of tasks separated into sub-stages. The first four phases entailed designing, pilot testing, and modification of the treatments, while phase 5 included a phase II clinical trial with embedded qualitative research to evaluate the interventions and trial processes. The intervention design was informed by formative qualitative research with end-users. More specifically, we conducted 15 in depth semi-structured interviews with people at risk of type 2 diabetes to explore their views (acceptability and perceived utility) about the potential role of mHealth interventions for diabetes prevention, understand how a mHealth intervention may address the unmet needs of people with prediabetes for healthy lifestyle behavior, and to identify patient-elicited recommendations about how to optimize its impact. In addition, we conducted 15 in depth semi-structured interviews with primary care doctors and nurses to explore the acceptability, perceived usefulness, and suggestions regarding an educational co-intervention targeting healthcare professionals. All the interviews were audio-recorded, transcribed and analyzed using thematic analysis (17).

The PREDIABETEXT co-intervention targeted to people at risk of type 2 diabetes was then developed thought a number of iterative workshops with a multidisciplinary team of primary care doctors and nurses, endocrinologists, nutritionists, sports scientists, psychologists, and pharmacists. Three patients were actively involved to ensure the content was appropriate and understandable. Two diabetes experts reviewed the pre-final draft of the intervention to ensure its comprehensiveness and alignment with current guidelines. The intervention content was framed in short text messages that could be delivered to patients mobile phones through the Balearic Islands Health Service messaging platform. Once the intervention was developed, we piloted it during one month with 21 people with prediabetes to examine its acceptability, relevance, and perceived impact. Following the pilot study, we conducted individual semi-structured interviews with 12 patients diverse in terms of socioeconomic level to explore their experience of using the system. The results were used to fine-tunning the intervention.

The PREDIABETEXT co-intervention targeted to primary healthcare professionals consisted in an educational intervention to raise awareness about the importance of T2DM prevention, increase knowledge about effective strategies for diabetes prevention, improve knowledge about brief counseling techniques, and enhance communication skills. The intervention was developed by members of the research team (endocrinologists, nutritionists, GPs, nurses, and the managers for the continuous education to primary care professionals in the Balearic Islands). The contents were made available through an online platform from the Mallorca Primary Healthcare Teaching Unit. We piloted this educational intervention with ten primary care and nurses, who received the intervention contents and completed an *ad-hoc* questionnaire about its usefulness and suggestions for improvement. The intervention was then refined based on their feedback.

### Study design

To assess the impact of PREDIABETEXT, we designed a six-month, three-arm, cluster randomized, clinical trial (NCT05110625). A detailed description of the trial protocol is available elsewhere. Briefly, participating primary healthcare professionals (physicians and nurse) were randomized by computer-generated random numbers to intervention A group (patient text messaging intervention), intervention B group (patient text messaging intervention + providers online education intervention), or control group (usual care). Selection criteria for both primary care patients and professionals is described in **Box 1**.

Box 1 **Selection criteria in the PREDIABETEXT trial**

Primary health care professionals

- We included general practitioners (GPs) and nurses from primary care centers located in Mallorca (Balearic Islands, Spain).

- We excluded those planning to relocate to a different center during the study period and those who declined to participate.

Patients

- We included adult population between the ages of 18 and 75 years registered in the patient list of one of the primary healthcare professionals recruited in the PREDIABETEX trial.

- We included patients with HbA1c 6% to 6.4% registered in the last three months; or with two consecutive values of fasting plasma glucose between 110–125 mg/dL, or both.

- We excluded patients with a registered diagnosis of type 2 diabetes; with a prescription of any type of antidiabetic medication; with a history of pregnancy during the previous 12 months; with no access to a mobile device to capable of receiving SMS, not able to read Spanish, or with a severe mental condition.

### Outcomes

The primary outcome of the study was HbA1c at 6 months follow-up. Secondary outcomes included various clinical, physiological, motivational, and behavioral measures.

### Recruitment strategy

To maximize an efficient use of constrained resources, we designed a novel, fully virtual strategy to recruit participants into the trial. This strategy involved two stages: 1) recruitment of healthcare professionals, and 2) recruitment of patients from the already recruited professionals.

In the first stage, eligible healthcare professionals received an email invitation to participate in the study, with an enclosed participant information sheet. Subsequently, we phoned them to offer additional information about the study (if needed), and to seek informed consent. All the informed consents were obtained telephonically, audio-recorded, and stored in a secure server. In the second stage, with support from the Information Service platform (see acknowledgments), we obtained a list of eligible patients from data extracted from the Balearic Islands primary care database. Computer-generated random numbers were used to assign recruited family physicians and nurses to one of three research arms. Their patients received invitations to participate in the trial and offered the intervention based on the healthcare professional arm allocation. concealment of allocations was carried out at the healthcare professional level (clusters). Through the text messaging platform owned by the Balearic Islands Health Service, we sent potential participants an SMS invitation to take part in the study to patients from healthcare professionals who had already consented to participate in our trial. Along a brief invitation, the SMS included a link to a webpage containing: detailed information about the study, the Patient Information Sheet, and a video in which representatives of healthcare professionals and patients who had participated in the development and initial evaluation stages of the intervention explained the study (see online **Appendix A**). After 48 hours, a research assistant contacted the individuals by phone to extend a formal invitation to participate in the study, provide any additional information, and record their informed consent (also audio-recorded over the phone). Potential participants could opt out to be reached by phone in case they requested it through a form in the study website. New blood tests were ordered to confirm the eligibility of individuals with a blood test result registered more than three months before consenting to participate.

### Data collection

Immediately after recruitment baseline data of the participants lifestyle behavior was collected via a phone semi-structured interview using validated instruments:

- Adherence to the Mediterranean diet (MEDAS questionnaire). A score <9 was categorized as low adherence, ≥9 good adherence (18).
- Physical activity levels (REGICOR Short form Physical Activity Questionnaire) (19). Participants were classified as not very active (<300 METs min/weak), active (600-1200 METs min/weak), and very active (>1200 METs min/weak).
- Sedentary behavior (physical activity questionnaire utilized for the Nurses’ Health Study) (20).
- Smoking behavior (adapted version of the Global Adult Tobacco Survey (GATS) questionnaire). Participants categorized as past smokers, never smokers, or current smokers (21).
- Alcohol consumption, quantified in grams based on a validated calculator (22).

Subsequently, patients were invited to visit with the research nurse, who conducted anthropometric measurements and collected blood samples. Patients could choose to have the visit in their primary care center or in the reference hospital.

- Blood pressure (BP): normal BP (Systolic BP <130 and/or Diastolic BP <85 mmHg), Prehypertension (Systolic BP 130 to 139 and/or Diastolic BP 85 to 89 mmHg), and Hypertension (Systolic BP ≥140 and/or Diastolic BP ≥90 mmHg) (23).
- Cardiovascular disease (CVD) was assessed using the REGICOR-Framingham risk equation, validated for the Spanish adult population aged 35-74 years (24). Participants were categorized as low risk (<5%) and moderate or high risk ≥5%.
- Insulin resistance was calculated using the Homeostasis model assessment (HOMA) (25).
- Abdominal obesity (AO) was determined as Waist Circumference (WC) >102 cm for men and >88 cm for women, according to WHO criteria. AO was defined by the Waist to Hip Ratio (WHR) as values > 0.90 for men and > 0.85 for women (26).

### Sample size estimation

The sample size calculation was designed to detect clinically relevant differences in HbA1c outcomes between intervention and control groups, not to identify subgroup differences at baseline. A total of 420 patients (140 per group) and 42 primary healthcare providers (10 patients per provider) were planned. This calculation assumed 80% power, an intraclass correlation coefficient of 0.04, a design effect of 1.36, and a 10% dropout rate. Detecting a difference of at least 0.3% in HbA1c (SD = 0.8%) was considered sufficient, based on previous research, to demonstrate statistically significant differences in diabetes incidence in a future phase III trial with extended follow-up.

### Statistical methods

We used descriptive statistics for reporting the number (percentage) for categorical variables, and mean (SD) for continuous variables. Chi-square test, independent t-test and ANOVA were used for between-group comparisons as needed, including *post-hoc* test (Bonferroni correction) for significant p-values. Nonparametric tests were used in non-normal variables (Kruskal-Wallis or Mann-Whitney U tests). All the statistical analysis was conducted using IBM SPSS Statistics for Windows, Version 25.0 (IBM Corp., Armonk, NY). P value of <0.05 was considered statistically significant.

## Results

### Description of the interventions

The co-intervention targeted to patients at risk of T2DM consisted in sending three weekly SMS short text messages supporting lifestyle change behavior (motivation, type of food, portions, physical activity, sedentary lifestyle, etc.). This intervention is based on the Behaviour Change Wheel, a framework for describing, designing and evaluating behavior change strategies (27). The messages are based on a total of 22 different well-established behavior change techniques, the most frequents ones being “Instruction on how to perform a behavior”, and “Health consequences” (28). Messages are personalized based on participants characteristics (BMI, alcohol drinking and smoking behavior, gender, and having or not internet access through their phones). Of the 71 messages delivered over the six months intervention period around 40% focused on healthy diet, 32% on physical activity, 12% on information about diabetes and diabetes prevention, and 10% on motivational strategies.

The intervention targeted to healthcare professionals consisted in a one-month online course about the management of patients with prediabetes at the primary healthcare level. Main topics addressed were: epidemiology, diagnostic criteria, motivational interviewing, treatment, and monitoring and follow up. This online educational intervention offered support of different activities—including solving clinical cases and watching clinical practice simulation videos.

### Recruitment and characteristics of the healthcare professionals

**Figure 1:**
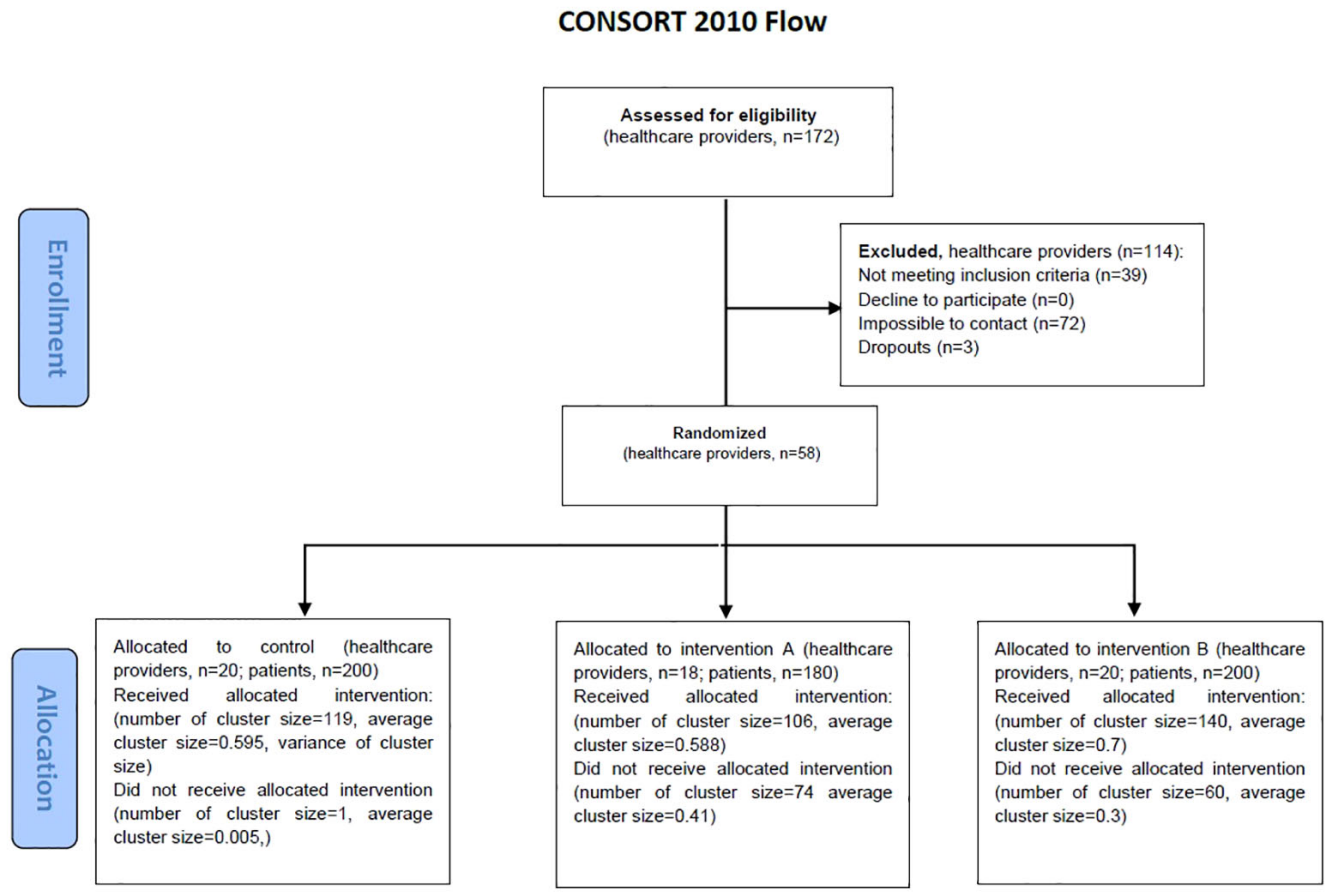
Participant flow diagram from enrolment to randomization.

Of 172 healthcare professionals who received an initial invitation by email, 133 confirmed meeting the eligibility criteria during phone interviews. Among them, 58 professionals (30 doctors; 28 nurses) from 16 centers were enrolled and completed the baseline telephone interview (recruitment rate: 58/133 = 43.6%). Most professionals (83%) were women [mean (SD) age 49.69 (10.15)]. 20 professionals were allocated to the control group, 18 to intervention group A, and 20 to intervention group B (**Figure 1**). Total mean (SD) number of patients assigned per healthcare professional was 1924.12 (387.31). No relevant differences were observed between the three groups (**Table 1**).

**Table 1:** Baseline characteristics of the healthcare professionals by intervention group.

PROFESSIONALS	Total (n=58)	Control (n=20)	Intervention A (SMS) (n=18)	Intervention B (SMS + training*) (n=20)	P value
Demographic characteristics					
Women, n (%)	48 (82.8%)	15 (75.0%)	17 (94.4%)	16 (80.0%)	0.26
Age (years), mean (SD)	49.69 (10.15)	48.35 (11.51)	51.78 (9.28)	49.15 (9.66)	0.56
Nurses, n (%)	28 (48.3%)	10 (50.0%)	9 (50.0%)	9 (45.0%)	0.93
Medical doctors, n (%)	30 (51.7%)	10 (50.0%)	9 (50.0%)	11 (55.0%)	
Years of working, mean (SD)	23.59 (9.22)	22.75 (10.02)	23.17 (8.56)	24.80 (9.29)	0.76
Professional works in a teaching center, n (%)	16 (28.1%)	8 (40.0%)	3 (17.6%)	5 (25.0%)	0.29
Mean (SD) number of patients assigned per healthcare professional	1924.12 (387.31)	1846.10 (342.95)	1996.50 (437.35)	1937.00 (386.94)	0.48
Mean (SD) number of patients recruited linked to healthcare professional	6.29 (3.60)	5.95 (3.72)	5.83 (3.47)	7.05 (3.66)	0.51

SMS, Short Message Service; SD, Standard Deviation.

*Health care professional training.

### Recruitment and characteristics of participating patients

Patient recruitment is shown in **Figure 1**. From data extracted from electronic health records, we identified an overall population of 7,116 patients potentially meeting the eligibility criteria described in **Box 1**. Their sociodemographic and clinical characteristics of the overall are available in Online **Appendix B**. We sent SMS recruitment invitations to a sample of 1,233 patients. Our recruitment team successfully phoned 1,056 patients, of which 975 confirmed meeting the eligibility criteria. A total of 534/975 (54.78%) accepted to participate in the trial, and provided informed consent. However, 67 were excluded because failed to attend their appointment with the research nurse; and 102 were excluded due to no longer meeting the eligibility criteria for prediabetes according to the baseline blood test results. Therefore, we successfully enrolled and collected full baseline data from 365/1056 invited over the phone, yielding a recruitment rate of 34.5%. Recruitment and baseline data collection lasted 27 weeks (from 18 November 2021 to 31 May 2022). Recruitment and baseline interviews cost 14,230€ (39€ per enrolled participant); and anthropometric and blood sample extraction and analysis 6,013€ (16.5€ per enrolled participant).

The characteristics of the enrolled participants are described in **Table 2**. Patients were allocated to intervention group A (n=106), intervention group B (n=140), and control group (n=119). The three groups were balanced for all sociodemographic and clinical characteristics, with the exception of DBP, which was substantially higher in the intervention A than in the control group (78.75 and 74.60, respectively).

**Table 2:** Baseline demographic, clinical, and lifestyle variables of the patients according to the three trial arms.

	total (n=365)	Control (n=119)	Intervention A (SMS) (n=106)	Intervention B (SMS + training) (n=140)	P value^$^
Demographic characteristics					
Women, n (%)	199 (54.5%)	69 (58%)	54 (50.9%)	76 (54.3%)	0.57
Age (years), mean (SD)	59.79 (9.75)	60.75 (9.77)	58.66 (9.60)	59.84 (9.83)	0.27
Clinical characteristics, mean (SD)					
Height (cm), mean (SD)	162.82 (9.45)	162.29 (9.70)	163.28 (9.49)	162.93 (9.24)	0.72
Weight (kg)	84.38 (18.61)	84.71 (21.73)	84.57 (16.63)	83.96 (17.22)	0.94
BMI (kg/m2), mean (SD)	31.72 (5.97)	31.98 (6.78)	31.67 (5.56)	31.54 (5.47)	0.12
WC (cm), mean (SD)	103.77 (13.10)	103.49 (15.77)	103.92(11.48)	103.89 (11.49)	0.82^#^
HC (cm), mean (SD)	110.06 (11.74)	110.93 (13.76)	109.35 (10.64)	109.87 (10.66)	0.65^#^
WHR	0.94 (0.08)	0.93 (0.09)	0.95 (0.07)	0.94 (0.07)	0.20^#^
SBP (mmHg), mean (SD)	133.35 (15.50)	130.78 (15.45)	135.22 (16.42)	134.10 (14.63)	0.07
DBP (mmHg), mean (SD)	76.34 (10.80)	74.60 (11.19)^a^	78.75 (11.04)^a,b^	75.97 (9.97)^b^	0.01
Glucose (mg/dl), mean (SD)	104.28 (13.22)	102.90 (12.20)	105.18 (13.03)	104.78 (14.16)	0.37
TG (mg/dl), mean (SD)	141.09 (94.63)	123.48 (54.17)	156.85 (118.26)	144.22 (99.79)	0.18^#^
Chol (mg/dl), mean (SD)	194.72 (38.53)	195.98 (40.39)	195.33 (34.78)	193.14 (39.83)	0.826
LDL (mg/dl), mean (SD)	117.68 (33.61)	121.62 (35.62)	116.88 (28.15)	114.83 (35.41)	0.09^#^
HDL (mg/dl), mean (SD)	49.70 (11.60)	50.22 (11.82)	48.63 (11.27)	50.07 (11.69)	0.532
TG/HDL	3.17 (3.58)	2.65 (1.61)	3.66 (3.67)	3.25 (4.57)	0.22^#^
Chol/HDL	3.97 (1.22)	3.98 (1.03)	4.16 (1.26)	3.82 (1.31)	0.17^#^
HbA1c (%), mean (SD)	6.13 (0.16)	6.13 (0.15)	6.13 (0.15)	6.15 (0.18) $	0.32^#^
Insulin level (U/ml), mean (SD)	17.77(15.55)	15.42(7.88)	18.80 (13.7)	18.76(20.87)	0.67^#^
HOMA, mean (SD)	4.86 (5.45)	3.89 (2.26)	4.87 (3.70)	5.58 (7.76)	0.74^#^
REGICOR, mean (SD)	4.04 (2.65)	5.11 (4.30)	3.98 (2.25)	4.26 (2.95)	0.58^#^
Categories of Framingham-REGICOR					
Low risk, n (%)	257 (72.4)	85 (73.3)	74 (70.5)	98 (73.1)	0.87
Moderate or high risk, n (%)	98 (27.6)	31 (26.7)	31 (29.5)	36 (26.9)	
Adherence to Mediterranean diet					
MEDAS_score, mean (SD)	7.52 (2.04)	7.41 (2.08)	7.56 (2.35)	7.58 (1.74)	0.783^#^
Low adherence, n (%)	246 (67.4%)	83 (69.7%)	65 (61.3%)	98 (70.0%)	0.285^*^
Good adherence, n (%)	119 (32.6%)	36 (30.3%)	41 (38.7%)	42 (30.0%)	
Physical activity level					
Not very active, n (%)	191 (52.4%)	69 (58.0%)	55 (51.9%)	67 (47.9%)	0.456^*^
Active, n (%)	83 (22.7%)	22 (18.5%)	23 (21.7%)	38 (27.1%)	
Very active, n (%)	91 (24.9%)	28 (23.5%)	28 (26.4%)	35 (25.0%)	
Total energy expenditure in physical activity (METs minutes/week), mean (SD)	1983.78 (2365.91)	1987.94 (2413.43)	1914.54 (2356.85)	2032.66 (2347.65)	0.55^#^
Sedentary lifestyle					
Daily hours a day watching television, mean (SD)	3.52 (1.78)	3.64 (1.79)	3.48 (1.96)	3.44 (1.62)	0.56^#^
Daily hours a day sitting in front of computer/mobile/tablet screen, mean (SD)	0.98 (1.55)	1.18 (1.80)	0.92 (1.42)	0.85 (1.39)	0.37^#^
Daily hours a day sitting in any means of transportation, mean (SD)	0.33 (0.67)	0.27 (0.36)	0.29 (0.65)	0.41 (0.85)	0.46^#^
Daily hours a day sitting, mean (SD)	4.57 (1.83)	4.84 (1.82)	4.45 (2.09)	4.42 (1.61)	0.10^#^
Smoking habit					
Past or never smoker, n (%)	293 (80.3)	95 (79.8)	93 (87.7)	105 (75)	0.04^*^
Current smoker, n (%)	72 (19.7)	24 (20.2)	13 (12.3)	35 (25)	
Alcohol use					
Alcohol units/week, mean (SD)	3.027 (7.188)	3.546 (7.476)	3.010 (7.073)	2.600 (7.044)	0.03^#^
Low-risk consumption, n (%)	346 (95.1%)	112 (94.1%)	100 (95.2%)	134 (95.7%)	0.83^*^
Hazardous or risky consumption, n (%)	9 (2.5%)	7 (5.9%)	5 (4.8%)	6 (4.3%)	

Data are expressed as mean (SD) or N (%).

BMI, Body Mass Index; WC, Waist Circumference; HC, Hip Circumference; WHR, Waist to Hip Ratio; SBP, Systolic Blood Pressure; DBP, Diastolic Blood Pressure; TG, Triglyceride; Chol, Cholesterol; LDL, Low-Density Lipoprotein; HDL, High-Density Lipoprotein; HbA1c, Glycated hemoglobin; HOMA, homeostasis model assessment.

^$^One way ANOVA.

^*^Chi Square.

^#^Kruskal-Wallis Test.

^a,b^ *Post Hoc* test.

The 365 recruited participants were representative of the overall sample of 7,116 potential participants in terms of gender (53.6% women in the overall sample vs 54.5% in the recruited sample), mean age (60.9 vs 59.8), total cholesterol (198.3 vs 194.7), HDL cholesterol (49.1 vs 49.7 mg/dl), LDL cholesterol (120.97 vs 117.7 mg/dl), triglycerides (147.4 vs 141.1 mg/dl), HbA1c (6.11% vs 6.13%) and blood glucose (104.5 vs 104.3 mg/dl). **Appendix C** shows the total Baseline sociodemographic and clinical characteristics of the patients enrolled in the PREDIABETEXT trial, by gender.

The lifestyle characteristics of the study participants are shown in **Table 2**. Notably, only 32.6% of individuals showed good adherence to the Mediterranean diet. Additionally, it was observed that only 47.6% of the study participants were physically active.

## Discussion

PREDIABETEXT, a novel, evidence-based, multifaceted, digital intervention to prevent type 2 diabetes, has been successfully designed following state-of-the-art methods with heavy involvement from end users through formative qualitative research. Baseline results from a pragmatic cluster randomized controlled trial to evaluate the efficacy of the PREDIABETEXT intervention shows that a virtual recruitment was a feasible and efficient strategy for recruiting 58 primary healthcare professionals and 365 patients at risk of developing T2DM from 16 primary care centers in Mallorca. The sample of recruited patients was representative of the overall population of people with prediabetes in Mallorca in terms of key sociodemographic and clinical characteristics. This representativeness strengthens the trial’s external validity, ensuring that findings are generalizable to broader populations. It also provides a solid foundation for assessing the intervention’s efficacy and safety by minimizing selection bias and allowing meaningful subgroup analyses based on clinical and demographic characteristics.

### Trial recruitment

Effective recruitment of research participants is essential for successful randomized controlled trials and remains one of the most challenging and labor-intensive aspects of conducting research. The success of the recruitment strategy in this trial not only ensured sufficient sample size but also balanced distribution across intervention arms, reducing bias and facilitating the robust evaluation of covariates’ effects on outcomes. This achievement underscores the trial’s capacity to meet its scientific and translational goals. It causes missed clinical trial deadlines, leads to increased costs, and consumes more time than any other aspect of clinical trials (29). To make a more efficient use of limited resources, in this trial we followed a fully virtual recruitment strategy. By the time we started inviting healthcare professionals to participate, the coronavirus crisis was still not over. Despite initial concerns about potential reluctance, the recruitment rates (43.6% and 32.1% for healthcare professionals and patients, respectively) were acceptable - successfully meeting the recruitment targets within our 6 months’ timeline. In comparison to similar studies, the recruitment rate for surgeons in a study involving patients with prediabetes was 14.8% and 3.9% for the patients (30). Another clinical trial study among patients with prediabetes reported a recruitment rate of 10.4% (31). Conversely, in a study targeting a multiethnic Asian population with prediabetes and using smartphone app-based lifestyle interventions, the recruitment rate for patients was notably higher at 52% (32). The higher recruitment rate observed in our study can be attributed to several factors. First, we actively involved healthcare professionals and patients in the design and development of our recruitment strategies. As observed in a recent systematic review, actively engaging patients can successfully improve trials recruitment and retention rates (33). Engaging patients in the design of the intervention through our qualitative interviews, as well as in the development of the recruitment materials (most notably in videos with patients and healthcare professionals explaining the trial and encouraging enrolment) could have been key driver of our success. Second, we provided comprehensive information to all team members involved in supporting the recruitment process. Additionally, our study implemented proactive measures, such as nurses visiting health centers for patient recruitment, and making multiple attempts to each potential participant before considering them for rejection.

A recent systematic review comparing study samples recruited with virtual versus traditional recruitment methods observed that, although virtual recruitment samples are recruited faster, they are slightly younger, have more women participants, and are split on enrolment of racial minorities as compared to comparator studies (34). In our study our recruited sample was very similar to the overall population of people with diabetes in terms of gender, age, blood glucose and lipid profile.

### Characteristics of the enrolled participants

The study population had a mean age of 60 years, and 54% of them were women. This gender distribution closely aligns with the overall proportion of women in Spain’s total population, which stands at 51% (35). Regarding BMI of the study participants, 88.5% of them had a BMI of 25 or higher. The prevalence of overweight or obesity in the study of Mexican-Americans patients with prediabetes by Vatcheva et al. was 86.9% which is very similar to our finding (36). In our study, hypercholesterolemia was observed in approximately 49% of women and 38% of men. In contrast, a 2012 study in Spain among individuals with prediabetes reported a lower prevalence of 16% for women and 11% for men (37). Both studies highlighted lower levels of cholesterol in men compared to their female counterparts. Additionally, hypertension was found in 36.6% of women and 37% of men participants, which is lower than the percentages reported in similar studies involving participants with prediabetes in Spain, where the prevalence was 61.6% and 72.3%, respectively (37, 38).

Evaluation of lifestyle characteristics of the patients showed that low adherence to the Mediterranean diet in our study participants was 67.4%. Similar to our finding in a cohort study among individuals with prediabetes, low adherence to the Mediterranean diet was reported in 73% of participants (39). Additionally, about 52.3% of our study participants were categorized as not very physically active. Although the method of assessing physical activity in a similar study in patients with prediabetes was different from our study, 50.6% of the participants did not have weekly physical activity (40). In another study in Spain, 55% of patients with prediabetes were physically inactive (39). Regarding alcohol drinking habits, 4.9% of our study participants exhibited hazardous or risky alcohol consumption. In contrast, a study in Spain reported a prevalence of alcohol consumption of 12.7% for women and 34.1% for men with prediabetes (37). The differences in the prevalence of alcohol consumption may be attributed to the use of different classification criteria.

The variations in baseline variables among our study participants and similar studies in prediabetes patients may be attributed to differences in criteria for defining prediabetes. For instance, in the study by Diaz-Redondo et al. in Spain, prediabetes was defined as having FPG levels between 100 and 125 mg/dl, and/or an HbA1c range from 5.7% (39 mmol/mol) to 6.4% (46 mmol/mol) in the preceding 6 months (37). Nevertheless, despite some differences, the overall similarity of most variables to other prediabetes studies enhances the generalizability of the findings of this clinical trial to a wide range of patients with prediabetes across different populations.

### Strength and limitations

Our study has a number of strengths. First, we developed an intervention following best-in-class recommendations for the development of complex interventions (29). By conducting formative qualitative research with end users (healthcare professionals and patients), we were able to identify the main barriers and challenges that a digital intervention should take into consideration to successfully promote lifestyle change behavior among people at risk of diabetes type 2. Second, an important strength of PREDIABETEXT is its scalability at a low cost, thus representing a sustainable strategy for healthcare systems. We opted for a low-intensity intervention in an effort to improve the very low participation and retention rates typically observed in higher intensity, face-to-face programs, such as the DPP. Third, the fact that the PREDIABETEXT intervention is delivered under real world conditions, using resources from the Balearic Islands health services (secure access to electronic health records, SMS platform, moodle platform for online training) has two important advantages: better estimation of the impact of the intervention in routine clinical practice, and that the intervention can be instantly transferred to the health service if deemed appropriate. Additional strengths of our study are the use of validated questionnaires and blinded outcome assessors (which supports the internal validity of our results), and the fact that the enrolled patients are representative of the overall population of people with prediabetes in the Balearic Islands (which supports the external validity).

Our study has also some limitations. First, the use of self-reported measures for physical activity and dietary habits could introduce information and recall bias. The use of objective measures, such as pedometers, could have contributed to overcome these limitations. Second, the exclusion of non-Spanish speaking individuals from minority groups may limit its generalizability. A potential limitation of our study is that we did not fully achieve the sample size initially calculated for the trial. While we successfully recruited a representative sample of patients and healthcare professionals, the slightly lower participant numbers may have reduced the statistical power to detect smaller effect sizes. However, the achieved sample remains robust for examining the primary outcomes and provides valuable insights into the feasibility and impact of the intervention under real-world conditions. Our study may have failed to enroll hard-to-reach groups, as a key entry criterion implied having a recently recorded test result. Socially vulnerable groups are at higher risk of developing type 2 diabetes, but at the same time are less likely to visit their primary care center. Future trials on this area should design and implement recruitment strategies more suitable to better engage harder to reach groups.

## Conclusions

Prediabetic adults with a variety of demographic and lifestyle characteristics have been successfully enrolled in the PREDIABETEX trial. These participants exhibit a range of metabolic conditions such as obesity, hyperglycemia, hyperinsulinemia, and dyslipidemia, all of which are associated with an elevated risk of developing diabetes and CVD. It is noteworthy that this study differs from most health interventions, as it focuses on implementing preventive interventions in individuals with prediabetes, a high-risk population for diabetes and its associated complications. The findings of this trial might have significant implications for primary care physicians, healthcare professionals, and other stakeholders in T2DM prevention and management. Further research is needed to assess the intervention’s long-term sustainability, scalability, and potential cost-effectiveness.

## Data Availability

The raw data supporting the conclusions of this article will be made available by the authors, without undue reservation.
