# Corrigendum: Describing the initial results of a pragmatic, cluster randomized clinical trial to examine the impact of a multifaceted digital intervention for the prevention of type 2 diabetes mellitus in the primary care setting: intervention design, recruitment strategy and participants’ baseline characteristics of the PREDIABETEXT trial

**DOI:** 10.3389/fendo.2025.1604990

**Published:** 2025-06-04

**Authors:** Sofía Mira-Martínez, Rocío Zamanillo-Campos, Narges Malih, Maria Antonia Fiol-deRoque, Escarlata Angullo-Martínez, Rafael Jimenez, Maria Jesús Serrano-Ripoll, José Iván Oña, Aina M. Galmes-Panades, Rocío Gómez-Juanes, Jadwiga Konieczna, Jerónima Miralles, Dora Romaguera, María Clara Vidal-Thomas, Joan Llobera-Canaves, Mauro García-Toro, Elena Gervilla-García, Catalina Vicens, Oana Bulilete, Juan José Montaño, Margalida Gili, Miquel Roca, Antoni Colom, Ignacio Ricci-Cabello

**Affiliations:** ^1^ Health Research Institute of the Balearic Islands (IdISBa), Palma de Mallorca, Spain; ^2^ Research Group in Primary Care and Promotion-Balearic Islands Community (GRAPP-caIB), Palma de Mallorca, Spain; ^3^ Primary Care Research Unit of Mallorca, Balearic Islands Health Service, Palma, Spain; ^4^ Research Network on Chronicity, Primary Care, and Health Promotion (RICAPPS), Instituto de Salud Carlos III (ISCIII), Madrid, Spain; ^5^ Statistical and Psychometric Procedures Applied in Health Sciences (PSICOMEST), Health Research Institute of the Balearic Islands (IdISBa), Palma de Mallorca, Spain; ^6^ Department of Psychology, University of the Balearic Islands (UIB), Palma, Spain; ^7^ Dra. Teresa Piqué Primary Health Care Center, Balearic Health Service, Palma, Spain; ^8^ Research Group on Nursing, Community & Global Health, Health Research Institute of the Balearic Islands (IdISBa), University Hospital Son Espases (HUSE), Palma, Spain; ^9^ Physical Activity and Sport Sciences Research Group (GICAFE), Institute for Educational Research and Innovation (IRIE), University of the Balearic Islands, Palma, Spain; ^10^ CIBER of Physiopathology of Obesity and Nutrition (CIBEROBN), Instituto de Salud Carlos III, Madrid, Spain; ^11^ University Institute of Health Science Research (IUNICS), University of the Balearic Islands, Palma, Spain; ^12^ Department of Medicine, University of the Balearic Islands, Palma, Spain; ^13^ Research Group on Nutritional Epidemiology & Cardiovascular Physiopathology (NUTRECOR), Health Research Institute of the Balearic Islands (IdISBa), University Hospital Son Espases (HUSE), Palma de Mallorca, Spain; ^14^ Son Serra-La Vileta Primary Health Care Center, Balearic Health Service, Palma, Spain; ^15^ Department of Geography, University of the Balearic Islands (UIB), Palma de Mallorca, Spain

**Keywords:** prediabetic state, preventive health services, clinical trial, primary health care, patient recruitment

In the published article, there was an error in the author list, and authors “Alfonso Leiva, Miquel Bennasar-Veny, Aina Maria Yañez” were erroneously included. The author list has been updated.

In the published article, there was a mistake in the Funding statement. The correct funding statement appears below.

The author(s) declare that financial support was received for the research, authorship, and/or publication of this article. This study was funded by the Health Research Institute of the Balearic Islands (IdISBa) through the intramural project “SYN20/04” It was also supported by Instituto de Salud Carlos III (ISCIII) through the project RD21/0016/0009, co-financed with funds from Unión Europea – NextGenerationEU, which funds the actions of the Recovery and Resilience Facility. MAF-dR was funded by the Health Research Institute of the Balearic Islands (grant number FOLIUM19/05; funded by ITS-2019-003).

The authors apologize for these errors and state that this does not change the scientific conclusions of the article in any way. The original article has been updated.

