# Supplementary figures and images for: Describing the initial results of a pragmatic, cluster randomized clinical trial to examine the impact of a multifaceted digital intervention for the prevention of type 2 diabetes mellitus in the primary care setting: intervention design, recruitment strategy and participants’ baseline characteristics of the PREDIABETEXT trial
Source: Front Endocrinol (Lausanne). 2025 Mar 31;16:1524336. doi: 10.3389/fendo.2025.1524336 (PMC11994424; doi:10.3389/fendo.2025.1524336)

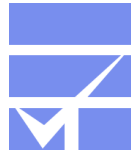

# CONSORT

TRANSPARENT REPORTING of TRIALS

## CONSORT 2010 Flow

Enrollment

Allocation

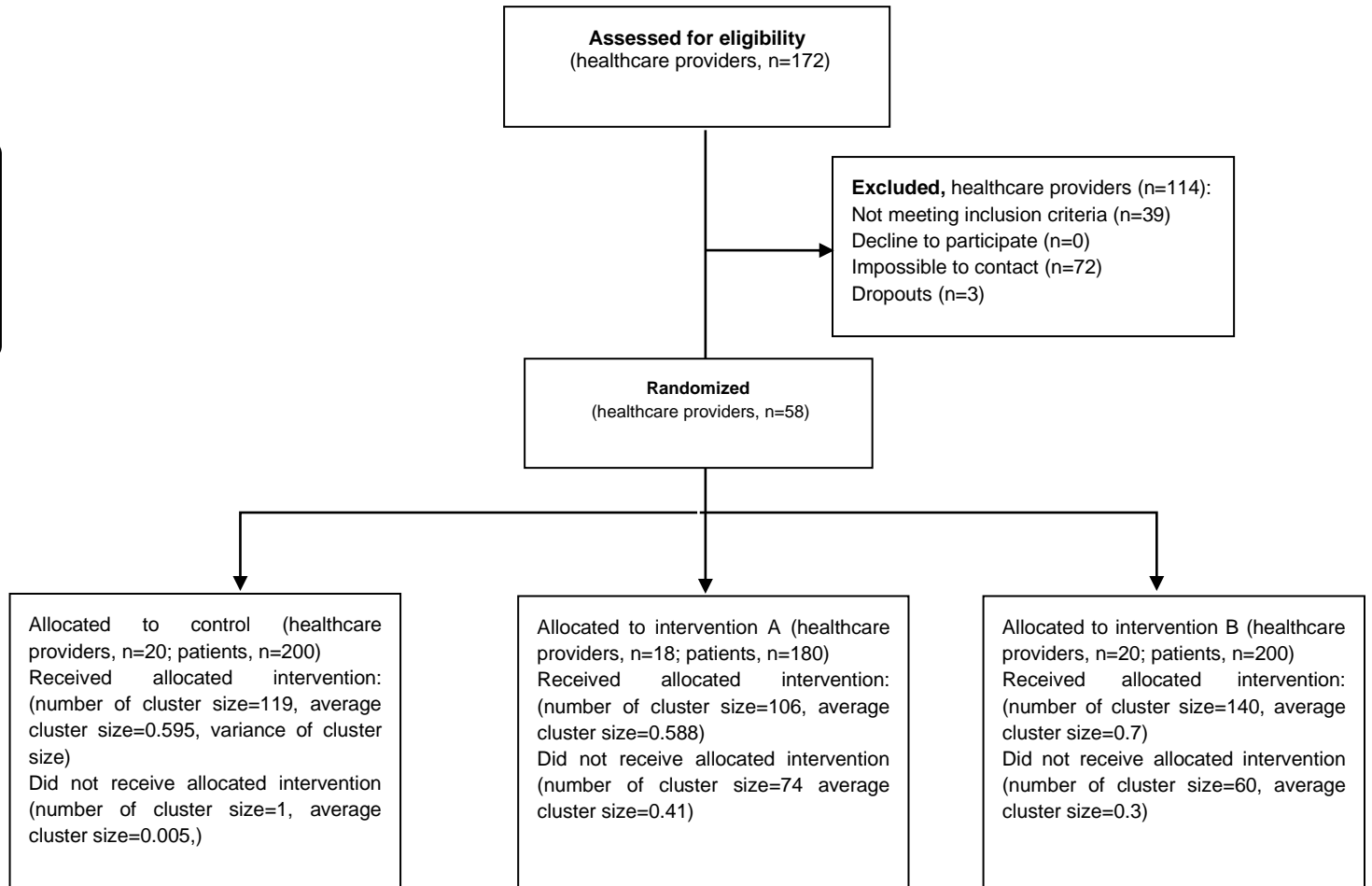

Supplement: Supplementary file 1 [file DataSheet1.pdf]
