# Supplementary material for: Describing the initial results of a pragmatic, cluster randomized clinical trial to examine the impact of a multifaceted digital intervention for the prevention of type 2 diabetes mellitus in the primary care setting: intervention design, recruitment strategy and participants’ baseline characteristics of the PREDIABETEXT trial
Source: Front Endocrinol (Lausanne). 2025 Mar 31;16:1524336. doi: 10.3389/fendo.2025.1524336 (PMC11994424; doi:10.3389/fendo.2025.1524336)
Supplement: Supplementary file 2 [file DataSheet2.docx]

Online Appendix B. Sociodemographic and clinical characteristics of the overall sample of potentially eligible participants for the PREDIABETEXT trial (n=7116)

|  | Overall population (n=7,116) | Enrolled in the PREDIABETEXT trial (n=365) |
| --- | --- | --- |
| Women, n (%) | 38.20 (53.68) | 199 (54.5) |
| Age (years), mean (SD) | 60.92 (9.90) | 59.79 (9.75) |
| HbA1c (%), mean (SD) | 6.11 (0.16) | 6.13 (0.16) |
| Chol (mg/dl), mean (SD) | 198.32 (40.76) |  |
| LDL (mg/dl), mean (SD) | 120.97 (35.24) |  |
| HDL (mg/dl), mean (SD) | 49.15 (12.37) |  |
| TG (mg/dl), mean (SD) | 147.38 (95.5) |  |

^Data are expressed as mean (SD) or N (%).^

^TG: Triglyceride, Chol: Cholesterol, LDL: Low-Density Lipoprotein, HDL: High-Density Lipoprotein, HbA1c: Glycated hemoglobin.^
