# Supplementary material for: Describing the initial results of a pragmatic, cluster randomized clinical trial to examine the impact of a multifaceted digital intervention for the prevention of type 2 diabetes mellitus in the primary care setting: intervention design, recruitment strategy and participants’ baseline characteristics of the PREDIABETEXT trial
Source: Front Endocrinol (Lausanne). 2025 Mar 31;16:1524336. doi: 10.3389/fendo.2025.1524336 (PMC11994424; doi:10.3389/fendo.2025.1524336)
Supplement: Supplementary file 3 [file DataSheet3.docx]

Online Appendix C. Baseline sociodemographic and clinical characteristics of the patients enrolled in the PREDIABETEXT trial, by gender.

The baseline sociodemographic, behavioural and clinical characteristics by gender are presented in Online Appendix 4. The mean (SD) age for women was 60.31 (9.82) years, while for men was 59.17 (9.66) years. Importantly, there was no statistically significant difference observed between the two groups (p=0.21, Mann-Whitney Test). Table 3 provides a comprehensive comparison of clinical baseline characteristics between the two gender groups.

Abdominal obesity, as determined by WHR, was observed in 91% of men participants and based on WC 71.7% of men had abdominal obesity. Regarding lifestyle characteristics, no significant difference was observed between men and women (p>0.05). Based on the categories of Framingham-REGICOR, a higher cardiovascular risk was noted among men participants (p<0.001).

Online Appendix C. Comparison of baseline clinical variables between men and women participants

|  | Total (n=365) | Women (n=199) | Men  (n=166) | *P* value^*^ |
| --- | --- | --- | --- | --- |
| Clinical characteristics, mean (SD) |  |  |  |  |
| Age (years), mean (SD) | 59.79 (9.75) | 60.31 (9.82) | 59.17 (9.66) | 0.21^#^ |
| Height (cm), mean (SD) | 162.82 (9.44) | 156.57 (6.56) | 170.31 (6.45) | <0.001 |
| weight (kg), mean (SD) | 84.38 (18.61) | 78.92 (19.17) | 90.92 (15.60) | <0.001^#^ |
| BMI (kg/m2), mean (SD) | 31.72 (5.97) | 32.05 (6.68) | 31.33 (4.99) | 0.94^#^ |
| WC (cm), mean (SD) | 103.77 (13.10) | 100.91 (14.02) | 107.18 (10.98) | <0.001^#^ |
| HC (cm), mean (SD) | 110.06 (11.74) | 111.57 (13.67) | 108.25 (8.58) | 0.15^#^ |
| WHR | 0.94 (0.08) | 0.90 (0.07) | 0.99 (0.06) | <0.001 |
| SBP (mmHg), mean (SD) | 133.35 (15.50) | 132.96 (16.10) | 133.80 (14.78) | 0.60 |
| DBP (mmHg), mean (SD) | 76.34 (10.80) | 76.57 (10.98) | 76.06 (10.59) | 0.65 |
| Glucose (mg/dl), mean (SD) | 104.28 (13.22) | 103.22 (11.74) | 103.30 (14.72) | 0.29^#^ |
| TG (mg/dl), mean (SD) | 141.09 (94.63) | 123.54 (56.24) | 162.41 (123.37) | 0.001^#^ |
| Chol (mg/dl), mean (SD) | 194.72 (38.53) | 199.61 (35.87) | 188.78 (41.13) | 0.008 |
| LDL (mg/dl), mean (SD) | 117.68 (33.61) | 121.07 (32.41) | 113.38 (34.69) | 0.03^#^ |
| HDL (mg/dl), mean (SD) | 49.70 (11.60) | 53.88 (10.92) | 44.64 (10.34) | <0.001^#^ |
| TG/HDL | 3.17 (3.58) | 2.41 (1.53) | 4.08 (4.90) | <0.001^#^ |
| Chol/HDL | 3.97 (1.22) | 3.67 (0.98) | 4.32 (1.37) | <0.001 |
| HbA1c, mean (SD) | 6.13 (0.16) | 6.13 (0.16) | 6.13 (0.15) | 0.60^#^ |
| Insulin level (µUI/ml), mean (SD) | 17.77 (15.55) | 16.69 (17.14) | 19.17 (13.23) | 0.08^#^ |
| HOMA, mean (SD) | 4.86 (5.45) | 4.85 (6.65) | 4.88 (3.43) | 0.16^#^ |
| REGICOR, mean (SD) | 4.04 (2.65) | 3.28 (1.74) | 4.95 (3.22) | <0.001^#^ |
| **Categories of Framingham-REG ICOR** |  |  |  |  |
| Low risk, n (%) | 257 (72.4) | 163 (84) | 94 (58.4) | <0.001^&^ |
| Moderate or high risk, n (%) | 98 (27.6) | 31 (16) | 67 (41.6) |  |
| BMI, n (%) |  |  |  |  |
| 18.5-24.9, n (%) | 42 (11.5) | 26 (13.1) | 16 (9.6) | 0.30^&^ |
| ≥25, n (%) | 323 (88.5) | 173 (86.9) | 150 (90.4) |  |
| Abdominal obesity by WC (men≥102cm, women≥88cm), n (%) | 285 (78.1) | 166 (83.4) | 119 (71.7) | 0.007^&^ |
| Abdominal obesity by WHR (men>0.90, women>0.85), n (%) | 302 (82.7) | 151 (75.9) | 151 (91) | <0.001^&^ |
| Hypercholesterolemia (Cholesterol (mg/dl)≥200), n (%) | 159 (44) | 97 (49) | 62 (38) | 0.03^&^ |
| Hyperglycaemia (Glucose (mg/dl)≥126), n (%) | 21(5.8) | 7 (3.5) | 14 (8.4) | 0.04^&^ |
| Hypertriglyceridemia (TG (mg/dl)≥200), n (%) | 55 (15.2) | 18 (9.1) | 37 (22.7) | <0.001^&^ |
| High risk HDL levels (men<40, women<50(mg/dl)), n (%) | 44 (32.4) | 40 (34.2) | 39 (37.1) | 0.73^&^ |
| **Blood pressure** |  |  |  |  |
| Normal blood pressure, n (%) | 129 (35.6) | 70 (35.5) | 59 (35.8) | 0.99^&^ |
| Prehypertension, n (%) | 100 (27.6) | 55 (28) | 45 (27.3) |  |
| Hypertension, n (%) | 133 (36.7) | 72 (36.5) | 61 (37) |  |
| **Physical activity status** |  |  |  |  |
| Not very active, n (%) | 191 (52.3) | 106 (53.3) | 85 (51.2) | 0.34^&^ |
| Active, n (%) | 83 (22.7) | 49 (24.6) | 34 (20.5) |  |
| Very active, n (%) | 91 (24.9) | 44 (22.1) | 47 (28.3) |  |
| **Adherence to Mediterranean diet** |  |  |  |  |
| Low adherence, n (%) | 246 (67.4) | 129 (64.8) | 117 (70.5) | 0.25^&^ |
| Good adherence, n (%) | 119 (32.6) | 70 (35.2) | 49 (29.5) |  |
| **Smoking habit** |  |  |  |  |
| Past or never smoker, n (%) | 293 (80.3) | 158 (79.4) | 135 (81.3) | 0.64^&^ |
| Current smoker, n (%) | 72 (19.7) | 41 (20.6) | 31 (18.7) |  |
| **UBEs^$^ risk level** |  |  |  |  |
| Low risk consumption, n (%) | 346 (95.1) | 193 (97) | 153 (92.7) | 0.06^&^ |
| Hazardous or risky consumption, n (%) | 18 (4.9) | 6 (3) | 12 (7.3) |  |

Data are expressed as mean (SD) or N (%).

BMI: Body Mass Index, WC: Waist Circumference, HC: Hip Circumference, WHR: Waist to Hip Ratio, SBP: Systolic Blood Pressure, DBP: Diastolic Blood Pressure, TG: Triglyceride, Chol: Cholesterol, LDL: Low Density Lipoprotein, HDL: High Density Lipoprotein, HbA1c: Glycated hemoglobin, HOMA: homeostasis model assessment

*Independent t-test

^#^Mann-Whitney U (non-parametric test)

^&^Chi-square test

^$^UBE: unidades de bebida estándar, https://www.fisterra.com/ayuda-en-consulta/calculos/cuantificacion-consumo-alcohol/
